# Differential roles of *BRCA1* and *BRCA2* in the DNA damage response revealed by isogenic V79 mutant cell lines

**DOI:** 10.1093/mutage/geag018

**Published:** 2026-04-24

**Authors:** Piyawan Chailapakul, Junko Maeda, Tomisato Miura, Takamitsu A Kato

**Affiliations:** Department of Environmental & Radiological Health Sciences, Colorado State University, Fort Collins, CO 80523, United States; Department of Environmental & Radiological Health Sciences, Colorado State University, Fort Collins, CO 80523, United States; Department of Risk Analysis and Biodosimetry, Institute of Radiation Emergency Medicine, Hirosaki University, Aomori 036-8564, Japan; Department of Environmental & Radiological Health Sciences, Colorado State University, Fort Collins, CO 80523, United States

**Keywords:** *BRCA1*, *BRCA2*, Chinese hamster cells

## Abstract

This study aimed to establish a novel *BRCA1* mutant cell line from Chinese hamster V79 cells and clarify the role of *BRCA1* in the DNA damage response (DDR) by comparison with a *BRCA2* mutant line, V-C8. Using CRISPR/Cas9 editing, we generated a hypomorphic *BRCA1* mutant, designated B1-21, carrying a 27-bp in-frame deletion in exon 4. This mutation deletes nine amino acids within the RING domain. B1-21, V79, and V-C8 cells were analyzed for DDR phenotypes. Both mutants showed impaired RAD51 foci formation, defective homologous recombination repair, and increased sensitivity to DNA-damaging agents. B1-21 cells were particularly sensitive to camptothecin and the PARP inhibitor NU1025, whereas V-C8 cells showed higher sensitivity to etoposide, cisplatin, mitomycin C, and bleomycin. Although γH2AX and FANCD2 focus responses were similar between V79 and B1-21, RAD51 recruitment was only partially reduced in B1-21 and completely absent in V-C8. B1-21 also displayed chromosomal instability (19–20 chromosomes), while V79 and V-C8 maintained a stable karyotype. After gamma irradiation, V-C8 cells accumulated substantially more chromatid-type aberrations and retained unrepaired chromatin longer than B1-21. Neither mutant showed normal RAD51 foci formation, radiation-induced sister chromatid exchange or an effective G2/M checkpoint arrest, unlike wild-type cells. Mitotic index measurements further confirmed checkpoint failure: V79 cells suppressed mitotic entry after irradiation, while B1-21 and V-C8 continued to enter mitosis, with V-C8 showing the most complete checkpoint breakdown. These findings indicate that partial disruption of the BRCA1 RING domain results in a hypomorphic phenotype with impaired homologous recombination, defective checkpoint control, and enhanced genotoxic sensitivity. The isogenic *BRCA1* and *BRCA2* mutant V79 lines offer a valuable model for dissecting DDR pathway differences and developing mutation-specific therapeutic strategies targeting *BRCA*-mutant cancers.

## Introduction

The DNA damage response (DDR) comprises a complex network of cellular pathways that detect DNA lesions, transduce damage signals, and coordinate DNA repair to preserve genomic integrity. Among various types of DNA damage, double-strand breaks (DSBs) are particularly deleterious. If left unrepaired or misrepaired, DSBs can lead to chromosomal rearrangements, genomic instability, and ultimately tumorigenesis [1, 2]. The tumor suppressor genes *BRCA1* (breast cancer susceptibility gene 1) and *BRCA2* (breast cancer susceptibility gene 2) are critical components of the high-fidelity homologous recombination (HR) repair pathway, which is essential for precise DSB repair. Germline mutations in either gene compromise HR efficiency, conferring a high lifetime risk of breast, ovarian, and other cancers [3]. In breast cancers, germline mutations in *BRCA1* and *BRCA2* account for ~5%–10% of overall cases [4], whereas in ovarian cancers, the frequency ranges from 5% to 15% [5]. Despite their shared role in HR, *BRCA1* and *BRCA2* perform distinct molecular functions [6]. *BRCA1* acts upstream, regulating DNA end resection, chromatin remodeling, and checkpoint activation [7]. In contrast, *BRCA2* functions downstream by recruiting and stabilizing RAD51 at DSB sites to form the nucleoprotein filament that mediates strand invasion during HR [8].

The Chinese hamster V79 cell line has been widely used in radiation biology and DNA repair studies due to its stable karyotype, ease of culturing, and conservation of key DNA repair genes. Within this system, V-C8, a *BRCA2*-deficient derivative of V79 with biallelic *BRCA2* mutations, has been a powerful model for elucidating *BRCA2* functions [9–11]. V-C8 cells exhibit hypersensitivity to DNA-damaging agents, such as mitomycin C and ionizing radiation, defective RAD51 focus formation, and a high frequency of chromosomal aberrations. However, a comparable isogenic model of *BRCA1* deficiency has remained elusive, primarily due to the essential role of *BRCA1* during development. Indeed, complete *BRCA1* knockout is embryonically lethal in mice [12, 13], and most clinically relevant *BRCA1* mutations are frameshift insertions/deletions or missense mutations that produce truncated, non-functional, or hypomorphic proteins, often leading to partial rather than complete loss of function [3, 14]. Interestingly, the V79 cell line carries a p53 mutation [15], which enables it to tolerate the loss of essential gene functions. Previously, we successfully generated hypomorphic ATR mutant clones in this system and characterized their DDR phenotypes [16]. Based on this rationale, we applied CRISPR/Cas9 editing to generate a *BRCA1*-targeted V79 cell line and isolate a viable hypomorphic *BRCA1* mutant harboring an in-frame deletion in the RING (Really Interesting New Gene) domain on its N-terminus [17].

In this study, we directly compared this novel *BRCA1* mutant line B1-21 to wild-type V79 and *BRCA2*-deficient V-C8 cells to delineate the distinct and overlapping roles of *BRCA1* and *BRCA2* in DNA repair, checkpoint activation, and chromosomal stability under genotoxic stress. Our findings offer insights into the functional divergence of these two tumor suppressors and provide a foundation for mutation-specific therapeutic strategies.

## Materials and methods

### Cell culture

Chinese hamster lung-origin V79 cells, and the *BRCA2* mutant (V-C8) [9], were obtained from Dr Joel Bedford at Colorado State University (Fort Collins, CO, USA). The cells were cultured in Alpha-MEM (Thermo Fisher Scientific, Waltham, MA, USA) supplemented with 10% heat-inactivated fetal bovine serum (Sigma-Aldrich, St. Louis, MO, USA), 1% antibiotics (Anti-Anti; Invitrogen, Grand Island, NY, USA), and maintained in a 37°C incubator with 5% CO_2_ and humidity. For the CRISPR-mediated mutation of *BRCA1* in Chinese hamster cells, the *BRCA1* Double Nickase plasmid (h) (Cat no. sc-400093-NIC; Santa Cruz Biotechnology, Dallas, TX, USA) was transfected into V79 cells, following previously established protocols [16]. After selection with puromycin for 6 days, single-cell clones were isolated. Reverse transcription PCR was performed using the forward primer GGACCTAGTGCTTTCCGAGC and the reverse primer GGTAGCCCACACTCTGGATG, followed by Sanger sequencing via GENEWIZ (South Plainfield, NJ, USA) to confirm the mutation. The NCBI Reference Sequence of the Chinese hamster *BRCA1* gene was obtained from GenBank accession number XM_007645166.4. To assess basic cellular phenotypes, comparisons were made based on cell doubling time and plating efficiency using data from at least three independent experiments. For evaluating cell doubling time, 10,000 cells were plated into each well of a 12-well plate, and cell numbers were counted at four different time points (24, 48, 72, and 96 h). An exponential growth equation was used to calculate the cell doubling time. For evaluating cell plating efficiency, 100 cells were seeded into a P35 dish. After seven days of incubation, colonies were fixed, stained, and scored to assess clonogenicity.

### Chemicals

PARP inhibitor olaparib was obtained from Calbiochem (San Diego, CA, USA). Topoisomerase I inhibitor camptothecin, topoisomerase II inhibitor etoposide, DNA replication inhibitor hydroxyurea, DNA crosslinking agent cisplatin, alkylating agent methylmethane sulfonate (MMS), and DNA DSB-inducing agent bleomycin were obtained from Sigma-Aldrich. ATM inhibitor (KU55933) [18], ATR inhibitor (VE-821) [19], DNA-PK inhibitor (NU7441), and PARP inhibitor (NU1025) were acquired from Selleck Chemicals. Mitomycin C (MMC) was obtained from Funakoshi (Tokyo, Japan).

### Irradiations

Gamma-ray irradiation was performed using a ^137^Cs gamma-ray irradiator (J.L. Shepherd Model Mark I-68 nominal 6000 Ci, J.L. Shepherd, Carlsbad, CA, USA) at a dose rate of 2.5 Gy/min. UV-C irradiation was carried out at a wavelength of 254 nm using Phillips germicidal UV-C lamps (Phillips, Andover, MA, USA) with a fluence rate of 1 W/m^2^, as described previously [20]. Irradiation doses were selected based on the sensitivity and optimal detection of biologically relevant endpoints. Low doses of gamma radiation are particularly suitable for highly sensitive assays; with dose of 1 Gy can be used to quantify accurate assessment of DNA damage and repair dynamics without signal saturation or excessive cytotoxicity such as for foci formation, chromosome aberration analysis, and mitotic index (MI) measurements. In the context of UV-C exposure, 5–20 J/m^2^ of UV-C exposure is sufficient to induce measurable biological responses from significant amount of bulky DNA adducts in comparable sensitivity ranges. A moderate dose of 10 Gy was used for cell cycle analysis, as it induces a robust DNA damage checkpoint response and clear G2/M accumulation. For constant field gel electrophoresis (CFGE), a high radiation dose of 40 Gy was administered to generate a detectable migration pattern and sufficient fragmentation of DNA DSBs.

### Colony formation assay

The sensitivity of cells to DNA-damaging agents was evaluated using colony formation assays [21]. Each trypsinized cell was seeded to generate 300 colonies per dish and exposed to various dosages of different agents. The plated cells were incubated for 7 days to allow colony formation. After the incubation period, the cells were fixed with 100% ethanol and stained with 0.1% crystal violet. A colony was defined as containing more than 50 cells. Regression curves were generated from the survival fraction. IC_50_ values for the DNA-damaging agents and *D*_10_ values for Gamma rays and UV-C were calculated for each replicate using GraphPad Prism version 10.0.0, based on data from at least three independent experiments. Data are expressed as the mean ± standard error of the mean (SEM).

### Constant field gel electrophoresis

DNA DSBs were quantified using CFGE [22]. Cells were embedded in 0.5% agarose (Insert Agarose, FMC, Philadelphia, PA, USA) and irradiated with 40 Gy of gamma rays in an ice-cold medium (4 groups: unirradiated and after irradiation 0 , 1 h, and 3 h during recovery). After irradiation, cells were lysed on ice for 1 h using a solution containing 0.5 M EDTA, 0.01 M Tris, 2% Sarcosyl, and 0.2 mg/ml proteinase K (pH 8.0), followed by an overnight incubation at 50°C. Post-lysis, the samples were washed three times in TE buffer for 1 h each and then treated with 0.1 mg/ml RNase A (Sigma-Aldrich) at 37°C for 1 h. Electrophoresis was conducted at room temperature using a 0.6% pulse-field grade agarose gel (Bio-Rad, Hercules, CA, USA) and 0.5× TBE buffer, with a constant electric field of 0.6 V/cm for 40 h. The DNA in the gels was stained with ethidium bromide, and the intensities of the DNA retained in the “plug” versus the DNA released into the “lane” were quantified using a Chemi-Doc imager (Bio-Rad). The fraction of DNA released was calculated as the ratio of the intensity of DNA in the “lane”to the total DNA (plug + lane).

### Immunofluorescence analysis

Fluorescence immunocytochemistry was used to observe the formation of radiation-induced γH2AX, RAD51, and FANCD2 foci. After overnight incubation on P35 dishes, cells were exposed to 1 Gy of gamma rays at room temperature. To study the kinetics of γH2AX foci, samples were harvested at six time points: unirradiated and after exposure to 1 Gy of gamma radiation at 30 min, 1 h, 3 h, 6 h, and 24 h. Subsequently, the cells were fixed in 4% paraformaldehyde for 15 min, washed in phosphate-buffered saline (PBS), and permeabilized for 10 min in a solution of 0.5% Triton X-100 with 0.1% SDS in PBS. The cells were then blocked with 10% goat serum in PBS overnight at 4°C. Afterward, the cells were incubated with primary antibodies, diluted to 1:300 in a 10% goat serum PBS solution, including anti-γH2AX mouse monoclonal antibody (05-636, Millipore Sigma, Burlington, MA, USA), anti-RAD51 rabbit monoclonal antibody (8875, Cell Signaling Technology, Danvers, MA, USA), and anti-FANCD2 rabbit polyclonal antibody (NB100-182, Novus Biologicals, Centennial, CO, USA). Following 1 h of incubation at 37°C, the cells were washed with PBS and then incubated with Alexa 488-conjugated anti-mouse goat antibody and Alexa 594-conjugated anti-rabbit goat antibody (Invitrogen, Waltham, MA, USA), both diluted to 1:500 in a 10% goat serum PBS solution, for 1 h at 37°C. After this incubation, the cells were washed with PBS and mounted with a coverslip using DAPI in SlowFade (Invitrogen). Multi-dimensional fluorescence images were captured using a Zeiss Axiophot fluorescent microscope (Zeiss, Jena, Germany) equipped with a motorized z-stage and a CoolSNAP HQ2 Cooled CCD camera (Photometrics, Tucson, AZ, USA), along with QCapture Pro 6.0 software (Teledyne Q-Imaging, Surrey, BC, Canada). For foci analysis, at least 50 cells were examined per experiment.

### G1 chromosome aberration assay

Cells were synchronized into the G1 phase using the mitotic shake-off method [23]. Two hours after replating, cells were irradiated with 1 Gy of gamma rays and compared to non-irradiated controls. After 16 h, colcemid was added to collect metaphase cells. Cells were then trypsinized, treated with hypotonic KCl, and fixed in a 3:1 mixture of methanol and acetic acid solution [24]. Chromosome spreads were prepared and scored for chromosome- and chromatid-type aberrations. At least 50 metaphase spreads were evaluated per condition across three independent experiments.

### G2/M checkpoint arrest and G2 chromosome aberrations

Chromosome radiosensitivity was assessed using the G2 chromosomal assay, following an established method [25]. Log-phase cells were irradiated with either 1 Gy of gamma rays or 10 J/m^2^ of UV radiation, and the results were compared to unexposed controls. After a 30-min incubation, 0.1 μg/ml of colcemid was added for 1.5 h to arrest cells at metaphase. Cells were then trypsinized, treated with hypotonic KCl, and fixed in a 3:1 mixture of methanol and acetic acid solution [24]. Metaphase cells were spread onto glass slides, stained with 5% Giemsa solution, and examined under a Zeiss Axioskop microscope (Zeiss, Jena, Germany). MI was calculated from at least 100 cells, and chromatid breaks were scored in a minimum of 50 metaphase cells across three independent experiments. Cells were assumed to be in the G2 phase at the time of irradiation.

### G2 premature chromosome condensation assay

Exponentially growing cells were exposed to 1 Gy of gamma rays and treated with 50 nM Calyculin A (Sigma-Aldrich) for 30 min to induce premature chromosome condensation (PCC) [26]. Chromosome spreads were prepared and examined for chromatid aberrations at two time points: immediately after radiation (0 min) and 2 h post-treatment. Aberrations—including chromatid gaps, breaks, iso-breaks, and exchanges—were visualized using a Zeiss Axioskop microscope. A minimum of 50 PCC spreads were analyzed across three independent experiments.

### Sister chromatid exchange analysis

Cells were synchronized in the G1 phase using the mitotic shake-off method and then exposed to 1 Gy of gamma irradiation as a treatment or left untreated as controls. Following treatment, cells were cultured in a medium containing 10 μM 5-bromo-2′-deoxyuridine for two cell cycles (20 h) to allow incorporation into newly synthesized DNA. Colcemid was added during the final 6 h to arrest cells in metaphase. Chromosome spreads were prepared and subjected to differential staining to visualize sister chromatid exchanges (SCEs). For staining protocol, slides were incubated in a 5 μg/ml solution of Hoechst 33258 for 15 min, exposed to UV-B light for 15 min, and treated with a 2× SSC solution at 80°C for 30 min [27], followed by staining with 5% Giemsa. A minimum of 50 second-division metaphase spreads were examined per condition in each experiment, and SCEs were scored as an indicator of DNA replication-associated chromosomal instability.

### Flow cytometry

We observed the cell cycle distribution after exposing cells to 10 Gy of gamma rays from ^137^Cs. Following irradiation, the cells were fixed in 70% ethanol and stored at −20°C until analysis. For staining, a Propidium Iodide (PI) solution (10 μg/ml PI combined with RNase A) was used, and the samples were incubated at 37°C for 30 min. We analyzed 10,000 PI-stained cells using a CyAn™ ADP Analyzer and Summit v4.3.02 (Beckman Coulter, Loveland, CO, USA). The data were exported in flow cytometry standard format, and the histogram was evaluated using Modfit LT software (Verity Software House, Topsham, ME, USA) on Mac OS 9.

### Statistical analysis

Statistical analysis was performed to compare cellular and molecular responses among *BRCA1*-mutant (B1-21), *BRCA2*-mutant (V-C8), and wild-type (V79) cells. One-way analysis of variance (ANOVA) was used to assess cell doubling time, plating efficiency, and foci formation, with Tukey’s post hoc test for multiple comparisons. Mean IC_50_ and *D*_10_ values were compared using the Kruskal–Wallis test with post hoc analysis (Dunn’s test) among the BRCA-deficient clones compared to the wild-type. DDRs—including double-strand breaks and γH2AX foci kinetics—were analyzed using two-way repeated measures ANOVA. Chromosome aberrations, SCE, PCC, and MI were analyzed using two-way ANOVA to compare control and irradiated groups within each cell type, followed by Tukey’s post hoc test. Analyses were performed in GraphPad Prism version 10.0.0 (GraphPad Software, Boston, MA, USA), with *P*-values <.05 considered significant. All experiments included at least three biologically independent replicates.

## Result

### Characterization of basic cellular phenotype

**Figure 1:**
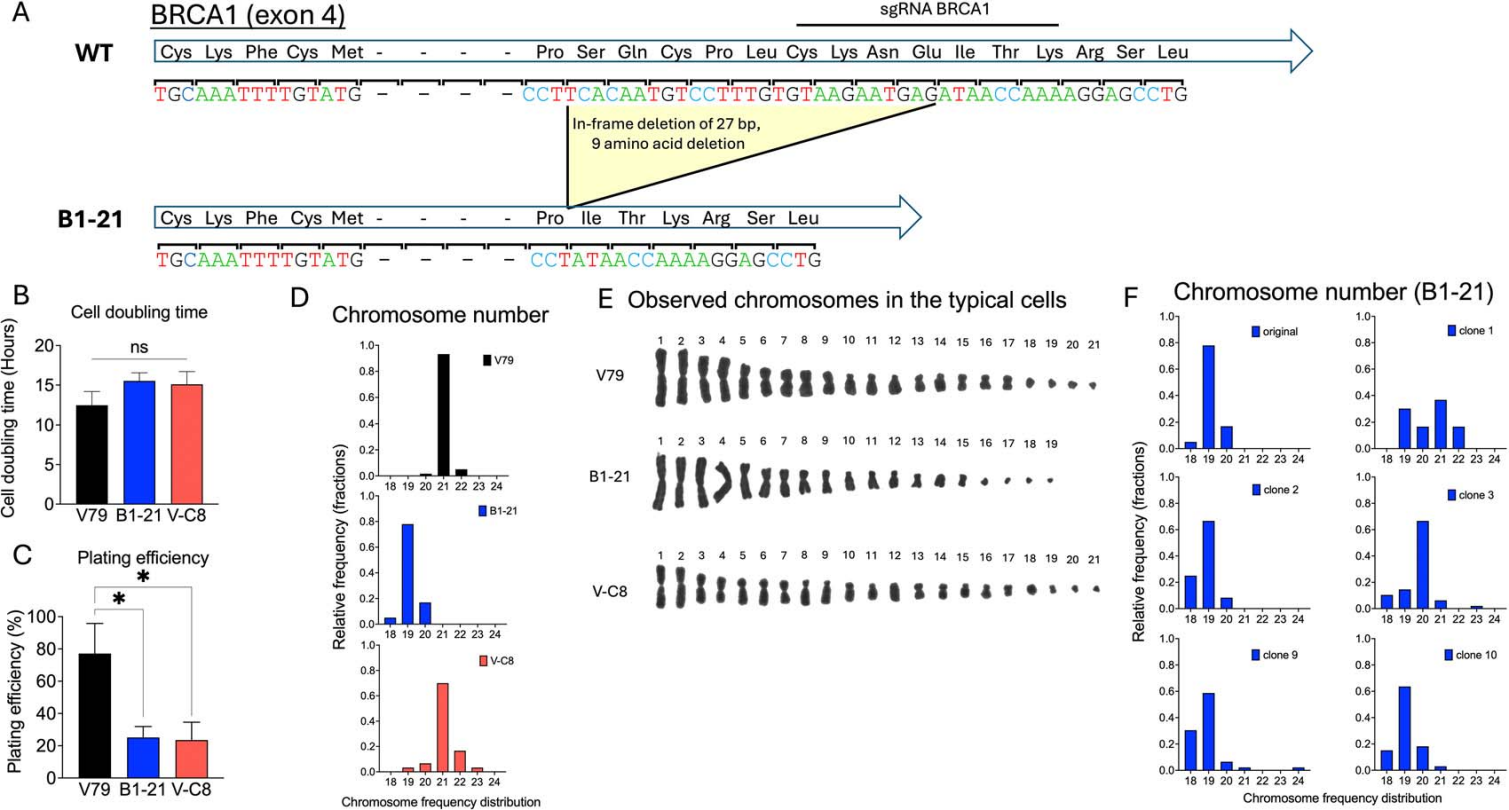
Characterization of V79 *BRCA1* mutant cells derived from CRISPR-Cas9 technology. (a) Sanger sequencing chromatograms confirmed targeted mutations in *BRCA1* in V79-derived clone. (b) Cell doubling time determined from exponential growth curves among V79, B1-21, and V-C8. (c) Plating efficiency assessed by colony formation following seeding at low density and incubation for 7 days among V79, B1-21, and V-C8. (d) Chromosome number distribution analysis in metaphase spread from V79, B1-21, and V-C8 cells. (e) Representative metaphase chromosome spreads from V79, B1-21, and V-C8. (f) Chromosome number distribution among independent B1-21 clones. Data represent mean ± standard errors of the means (SEM) from at least three independent experiments. An asterisk (*) indicates statistical significance (*P*-value <.05).

In order to create *BRCA1* mutant cells, *BRCA1* Double Nickase plasmids were transfected into V79 Chinese hamster cells using the CRISPR/Cas9 system. Post-transfection, a single-cell clone was isolated and subjected through DNA sequencing to verify the mutation. The resulting mutant clone, designated B1-21, contains a hypomorphic, in-frame deletion of 27 base pairs (c.175_201del) in exon 4 of *BRCA1*, which leads to the loss of nine amino acids. This mutation results in a mutated BRCA1 protein (p.Ser59_Glu67del), as shown in Fig. 1a.

The cell doubling time for V79 origin *BRCA1* and *BRCA2* mutants was 15.5 and 15 h, respectively, whereas parental V79 cells showed a doubling time of ~13 h (*P* = .35), as illustrated in Fig. 1b. The plating efficiency for the V79 wild-type cells was 77%, whereas it was 25% for the B1-21 and 24% for the V-C8, as shown in Fig. 1c. Statistical analysis demonstrates a significant difference between the wild-type and these mutants (*P* < .05). In the cytogenetic analysis, both V79 and V-C8 cells exhibited 21 chromosomes, whereas the B1-21 mutant was found to have only 19 chromosomes, as demonstrated in Fig. 1d and aligned in Fig. 1e. The chromosomal instability in B1-21 was confirmed with varying frequencies of chromosome numbers in re-cloned cells of 19 (B1-21 clone 2, 9, and 10), 20 (B1-21 clone 3) and 21 (B1-21 clone 1), as illustrated in Fig. 1f.

### Sensitivity to various DNA-damaging agents

**Figure 2:**
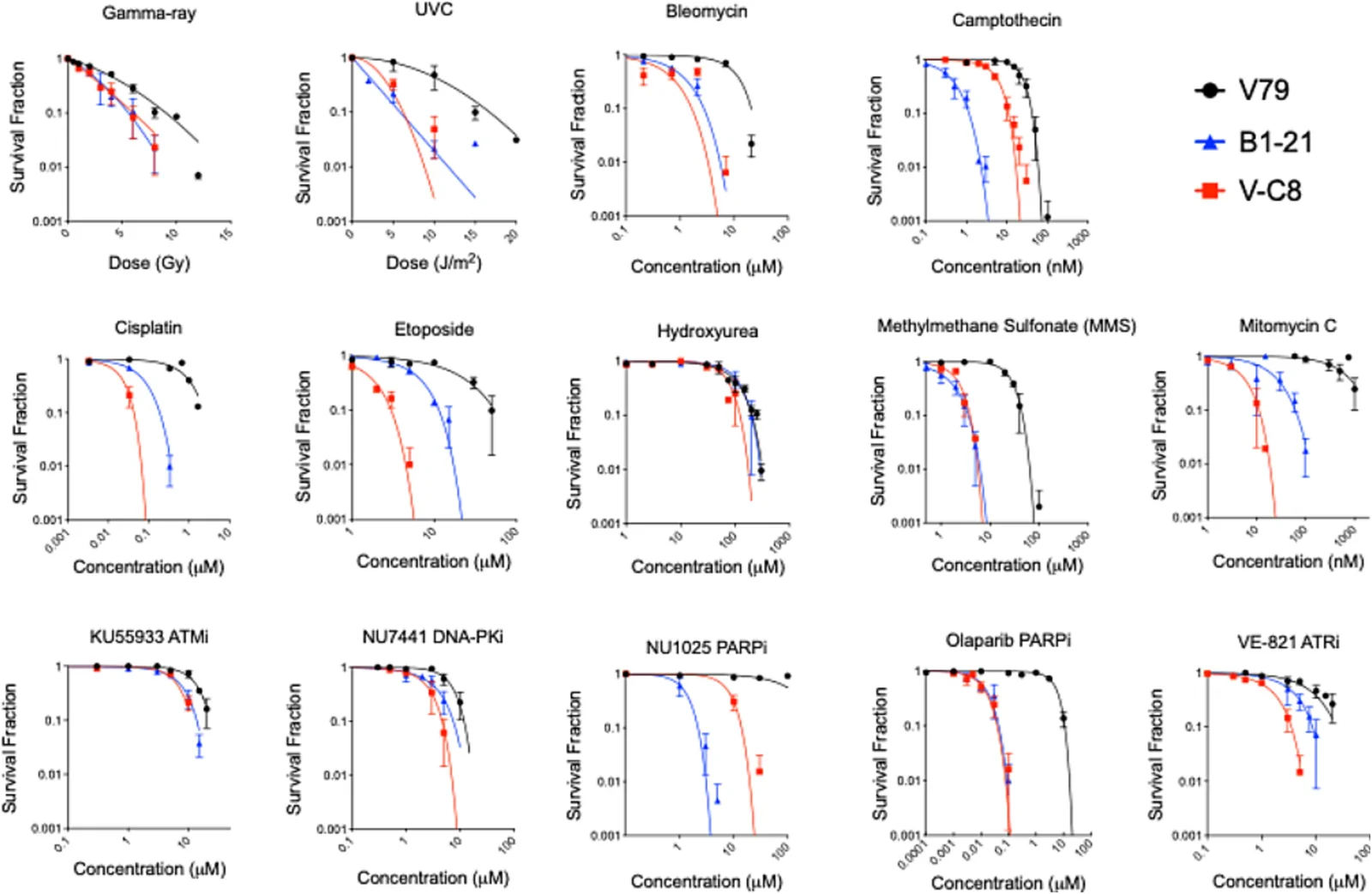
Sensitivity of V79 wild type, *BRCA1*-mutant (B1-21), and *BRCA2*-mutant (V-C8) cells to various DNA-damaging agents and DNA repair inhibitors. Cell survival was assessed using clonogenic assays following exposure to the indicated DNA-damaging agents (gamma rays and UV-C), or during exposure to DNA-damaging agents or inhibitors at varying doses and concentrations. Error bars indicate standard errors of the means.

We evaluated the sensitivity of three Chinese hamster cell lines—*BRCA1*-mutant (B1-21), *BRCA2*-mutant (V-C8), and wild-type (V79)—to various DNA-damaging agents using clonogenic survival assays, as shown in Fig. 2. The quantitative results are summarized in Table 1, which presents mean IC_50_ values ± SEM for various DNA-damaging agents and DNA repair inhibitors, and mean *D*_10_ values ± SEM for gamma rays and UV-C exposure. These values indicate differences in drug potency and the intrinsic resistance profiles of the respective cell lines.

**Table 1:** Cytotoxicity IC_50_ values for DNA-damaging agents/DNA repair inhibitors and *D*_10_ values for gamma ray and UV-C (mean ± SEM).

DNA-damaging agents	V79	B1-21	V-C8
**Sensitive to B1-21 (** $\frac{\mathbf{IC}_{\mathbf{50}}\mathbf{V}-\mathbf{C}\mathbf{8}\ }{\mathbf{IC}_{\mathbf{50}}\mathbf{B}\mathbf{1}-\mathbf{21}}$**> two-fold difference)**			
Camptothecin (nM)	24.4 ± 4.3	0.5 ± 0.1^*^	4.9 ± 0.4
NU1025 (μM)	54.9 ± 6.8	1.8 ± 1.0^*^	7.9 ± 1.2
**Sensitive to V-C8 (** $\frac{\mathbf{IC}_{\mathbf{50}}\mathbf{B}\mathbf{1}-\mathbf{21}\kern0.5em }{\mathbf{IC}_{\mathbf{50}}\mathbf{V}-\mathbf{C}\mathbf{8}\ }$**> two-fold difference)**			
Bleomycin (μM)	6.5 ± 1.3	1.0 ± 0.3	0.4 ± 0.2^*^
Cisplatin (μM)	0.5 ± 0.2	0.06 ± 0.006	0.02 ± 0.002^*^
Etoposide (μM)	14.7 ± 5.3	5.0 ± 0.7	1.3 ± 0.1^*^
Mitomycin C (nM)	604.2 ± 209.9	22.6 ± 7.7^*^	3.2 ± 1.1^*^
**Similar sensitivity (≤ two-fold difference)**			
Gamma ray (Gy)	8.8 ± 0.6	5.5 ± 1.2	5.5 ± 0.8^*^
UV-C (J/m^2^)	15.1 ± 1.5	6.2 ± 0.2^*^	8.3 ± 0.9
Hydroxyurea (μM)	85.7 ± 12.3	91.8 ± 13.4	59.0 ± 13.1
MMS (μM)	26.0 ± 2.3	1.2 ± 0.4^*^	1.7 ± 0.4
KU55933 (μM)	12.6 ± 1.5	6.8 ± 0.9	6.4 ± 0.3^*^
NU7441 (μM)	6.6 ± 1.1	3.1 ± 0.3	2.3 ± 0.6^*^
Olaparib (μM)	4.8 ± 0.8	0.008 ± 0.002^*^	0.02 ± 0.004^*^
VE-821 (μM)	8.2 ± 0.7	2.7 ± 0.9	1.4 ± 0.2^*^

The Kruskal–Wallis test with post hoc analysis (Dunn’s test) was conducted to assess the significance of the effects. An asterisk (^*^) denotes significance (*P*-value <.05) when comparing the mean IC_50_ values or *D*_10_ values among the BRCA-deficient clones compared to the wild-type cells.

Among the 14 agents tested, 13 showed statistically significant differences in IC_50_ and *D*_10_ values across the three cell lines, as determined by the Kruskal–Wallis test with post hoc Dunn’s analysis. This indicates altered drug responses in BRCA-deficient clones compared with wild-type cells, as shown in Table 1. A minimum two-fold difference in IC_50_ and *D*_10_ values was used as the threshold for biological significance. Sensitivity was determined based on the ratio between cell lines: if the IC_50_ (or *D*_10_) of V-C8 was more than two-fold higher than that of B1-21, B1-21 was considered more sensitive; conversely, if the IC_50_ (or *D*_10_) of B1-21 was more than two-fold higher than that of V-C8, V-C8 was considered more sensitive. Using this criterion, camptothecin and NU1025 showed greater sensitivity in B1-21 cells, whereas V-C8 cells were more sensitive to bleomycin, cisplatin, etoposide, and mitomycin C.

### DSB detection to repair kinetics

**Figure 3:**
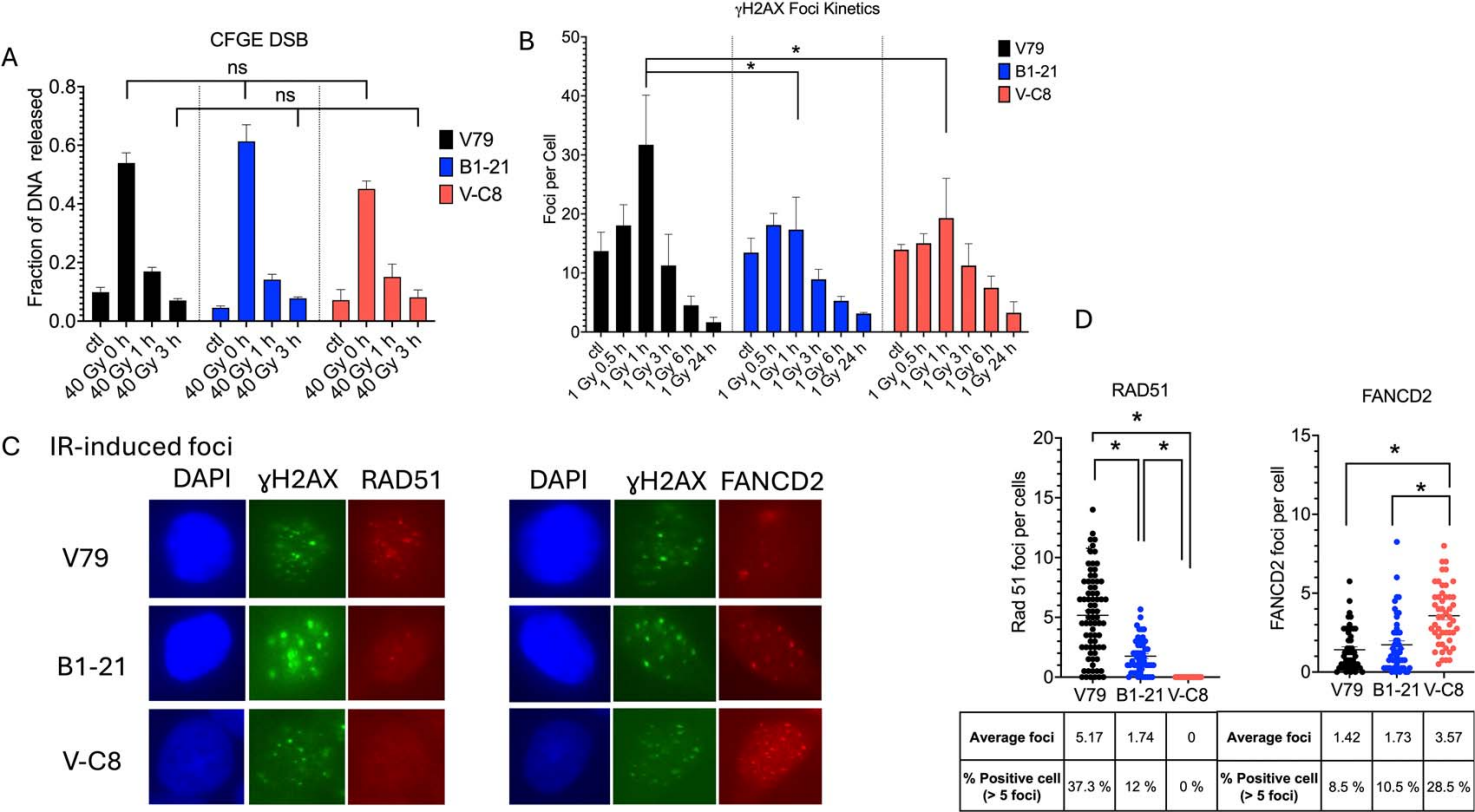
DNA damage responses among three cell lines. (a) CFGE was performed to assess DNA double-strand breaks following exposure to 40 Gy of gamma radiation. DNA fragmentation was analyzed immediately after 0, 1, and 3 h post-irradiation. (b) Kinetics of γH2AX foci formation after exposure to 1 Gy gamma-ray irradiation. Cells were fixed at indicated time points post-irradiation. (c) Immunofluorescence of IR-induced γH2AX, RAD51, and FANCD2 foci following 1 Gy of gamma radiation. Cells were fixed 1 h after exposure for foci formation. (d) Quantitative analysis for foci formation. Graphs represent the mean of each group, with error bars representing SEM. An asterisk (*) denotes significance (*P*-value <.05).

To assess DNA damage and detect DSBs following exposure to 40 Gy of gamma radiation, constant field gel electrophoresis was performed in three cell lines. All cell lines showed a marked increase in DSBs immediately after irradiation (0 h), followed by a decrease over time. Although DSB levels declined, there were no statistically significant differences in residual DSBs among the three cell lines at 3 h post-irradiation, as shown in Fig. 3a.

To further evaluate DNA repair kinetics, we analyzed the formation of γH2AX foci in these cell lines. All lines exhibited comparable baseline levels, averaging 10–11 foci per cell. High background foci number is associated with S-phase foci formation. Following exposure to 1 Gy of gamma radiation, γH2AX foci slightly increased at 30 min and peaked at 1 h, with V79 cells showing a significantly higher number of foci than the *BRCA*-mutant lines. At 3, 6, and 24 h post-irradiation, the number of foci declined across all groups. However, there was a slight difference in the number of foci between the *BRCA* mutants and the V79 cells at 24 h, with the *BRCA* mutants showing no significant increase (Fig. 3b). We also observed the radiation-induced formation of γH2AX, RAD51, and FANCD2 foci through immunofluorescence following exposure to 1 Gy of gamma irradiation, as illustrated in Fig. 3c. In V79 wild-type cells, all three markers were significantly induced and showed colocalization. The efficiency of HR repair was demonstrated by the formation of RAD51 foci, with an average of ~ 5.2 foci per cell, and 37% of the cells were classified as RAD51-positive (defined as having more than 5 foci per nucleus). In contrast, B1-21 cells displayed γH2AX and FANCD2 foci formation and colocalization similar to that of V79 cells; however, the RAD51 foci were markedly reduced to an average of 1.7 foci per cell, indicating impaired HR activity. The defect was more pronounced in V-C8 cells, where RAD51 foci were completely absent. On the other hand, V-C8 cells led to a significant increase in FANCD2 foci formation. About 29% of the V-C8 cells were positive for FANCD2 foci, which is substantially higher than the 8.5%–10.5% observed in both V79 and B1-21 cells (Fig. 3d).

### Chromosome aberration analysis

**Figure 4:**
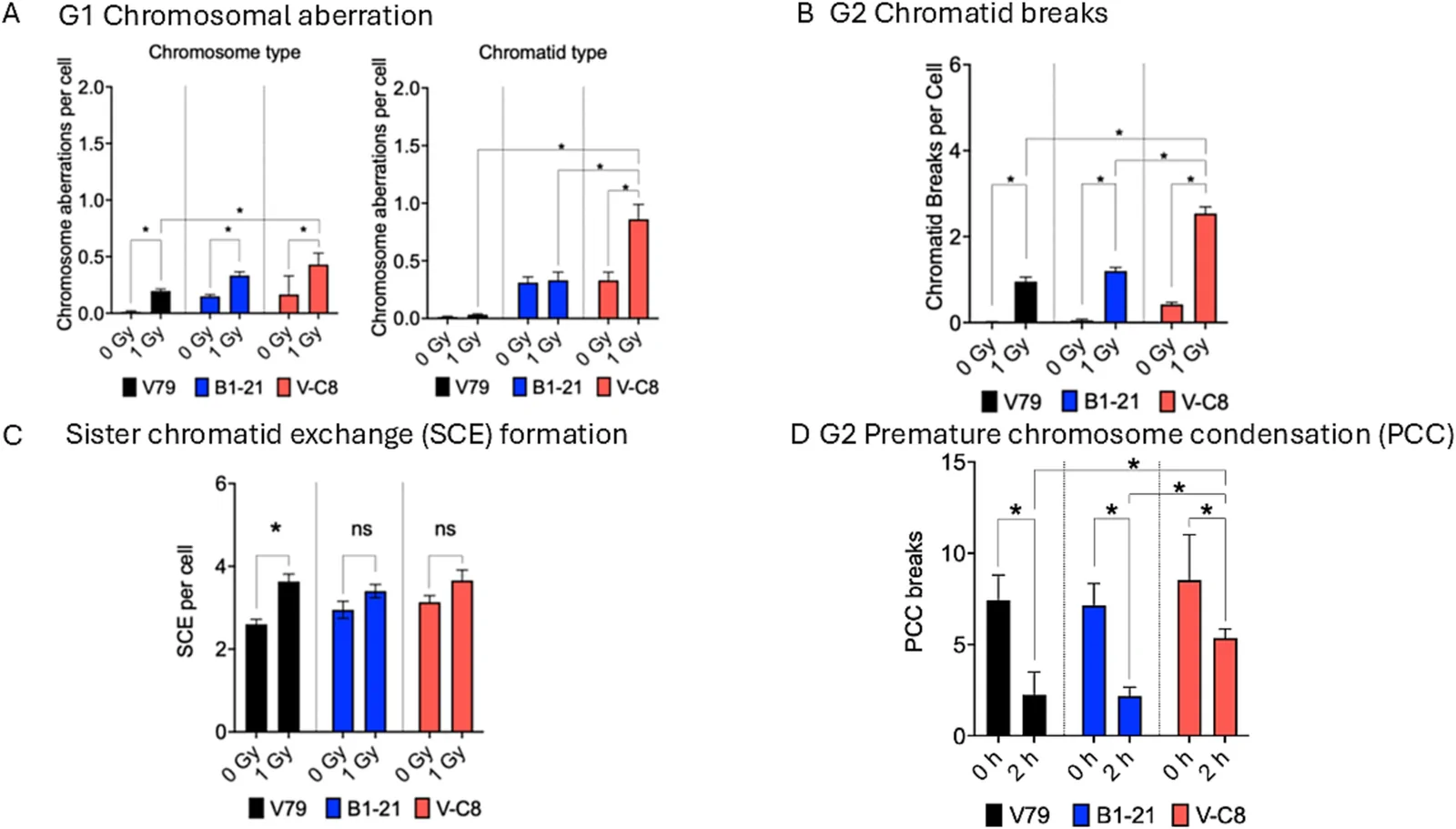
Chromosome aberration analysis induced by 1 Gy of gamma-ray irradiation (a) G1 chromosome aberration. G1 synchronized cells were irradiated with 1 Gy of gamma radiation, and the first post-irradiation metaphase cells were collected for analysis. (b) G2 chromosome aberrations (G2 chromatid breaks). Exponentially growing cells were irradiated with 1 Gy of gamma radiation, and colcemid was added at 30 min after irradiation, and metaphase cells were harvested at 120 min after irradiation. (c) Sister chromatid exchange (SCE) formation. G1 synchronized cells were irradiated with 1 Gy of gamma radiation, and the second post-irradiated metaphase cells were analyzed following BrdU incorporation and differential staining. (d) G2 PCC. Exponentially growing cells were irradiated with 1 Gy of gamma radiation, and Calyculin A was added either immediately or 2 h after irradiation to induce PCC. Graphs represent the mean of each group, with error bars representing SEM. Statistically significant differences were determined by two-way ANOVA and Tukey’s multiple comparison test. An asterisk (*) denotes significance (*P*-value <.05).

To examine chromosome abnormalities in G1 irradiated cells (Fig. 4a), we evaluated the frequency and types of radiation-induced chromosomal aberrations across the three cell lines. V79 cells exhibited low baseline levels of chromosomal aberrations, with very few chromatid-type aberrations and chromosome-type aberrations. Radiation-induced chromosomal aberrations were limited to chromosome-type aberrations. In contrast, both *BRCA1*-mutant (B1-21) and *BRCA2*-mutant (V-C8) cells showed spontaneous both chromatid-type and chromosome-type aberrations. Following 1 Gy gamma-ray irradiation, both mutants presented a comparable amount of radiation-induced chromosome-type aberrations. On the other hand, only V-C8 presented a notable increase in chromatid type aberrations compared to the other lines (*P* < .05). In the G2 phase irradiation (Fig. 4b), all three cell lines exhibited elevated levels of radiation-induced chromatid breaks, with V-C8 again showing the most pronounced effect among the other two cell lines (*P* < .05).

Analysis of SCE revealed that baseline SCE frequencies were similar among the three cell lines. However, after exposure to 1 Gy of gamma radiation, only V79 cells demonstrated a significant increase in SCE frequency (*P* < .05), whereas the *BRCA*-mutant cells showed no significant change (Fig. 4c). Additionally, we assessed chromatin integrity in interphase cells using the G2 PCC assay. All cell lines showed an increase in PCC breaks immediately following irradiation of 1 Gy of gamma rays, with the most substantial increase observed in V-C8 cells. While break frequency declined across all cell types after 2 h, V-C8 cells continued to exhibit a significantly elevated level of breaks compared to both V79 and B1-21 cells (*P* < .05). This persistent damage suggests that V-C8 cells may be defective in chromatin repair mechanisms during the G2 phase. In contrast, B1-21 cells displayed a PCC break profile similar to that of wild-type cells, indicating relatively preserved chromatin repair capacity (Fig. 4d).

### Cell cycle distribution and the effect of cell cycle arrest

**Figure 5:**
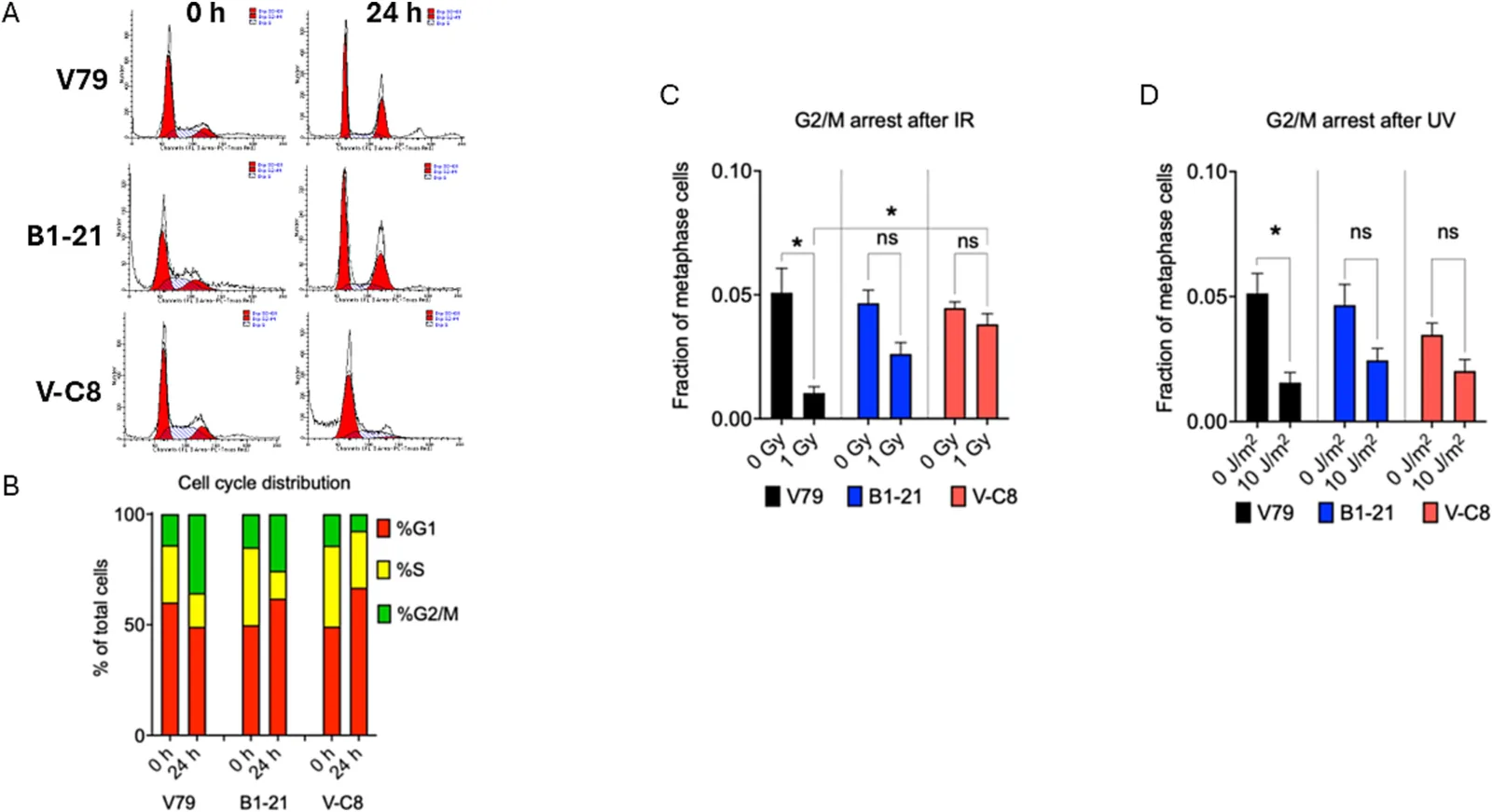
Analysis of the cell cycle and the effects of cell cycle arrest following DNA damage. (a) Representative flow cytometry histograms of DNA content measured by PI staining at 0 and 24 h after irradiation with 10 Gy of gamma radiation. (b) Quantification of cell cycle distribution (G1, S, and G2/M phases) from flow cytometry data. (c) MI following 1 Gy of gamma radiation. Cells were collected to assess G2/M arrest. Reduction of MI indicates IR-induced G2/M arrest. (d) Mitotic index (MI) 10 J/m^2^ of UV-C irradiation to evaluate UV-induced G2/M checkpoint activation. Graphs represent the mean of each group, with error bars representing SEM. Statistically significant differences were determined by two-way ANOVA and Tukey’s multiple comparison test. An asterisk (*) denotes significance (*P*-value <.05).

We analyzed cell cycle distribution by flow cytometry at 0 and 24 h after exposure to 10 Gy of gamma irradiation. At 0 h post-irradiation, all cell types (V79, B1-21, and V-C8) exhibited comparable profiles, with 50%–60% of cells in G1, 25%–35% in S, and 10%–15% in G2/M. By 24 h post-irradiation, V79 exhibited a reduction in the G0/G1 and S population and an accumulation in the G2/M phase (25%–35%), consistent with G2/M checkpoint activation. However, B1-21 cells also showed a slight increase in the G1 and G2/M phases. In contrast, V-C8 cells accumulated predominantly in the G1 phase, with no notable increase in G2/M phase, indicating a failure to activate the G2/M checkpoint, as shown in Fig. 5a and b.

To further assess the G2/M checkpoint functionality, we measured the MI following exposure to either 1 Gy of gamma radiation or 10 J/m^2^ of UV-C light. Thirty minutes after exposure, colcemid was added for 1.5 h before harvesting to arrest cells in metaphase, enabling quantification of mitotic entry. V79 cells displayed a significant reduction in MI following both gamma irradiation (*P* < .05) and UV-C exposure (*P* < .05), indicating an effective G2/M checkpoint. In contrast, B1-21 and V-C8 cells did not exhibit a significant reduction in MI after either gamma-ray (*P* = .26 and *P* = .98, respectively) or UV-C exposure (*P* = .22 and *P* = .86, respectively), as illustrated in Fig. 5c and d. These results suggest that while B1-21 cells retain partial checkpoint signaling as seen in flow cytometry, the arrest is not robust enough to fully suppress mitotic entry, similar to the checkpoint-defective phenotype of V-C8 cells.

## Discussion

In this study, we employed the CRISPR/Cas9 system to establish an isogenic V79 Chinese hamster cell model carrying a hypomorphic *BRCA1* allele, designated B1-21 (Fig. 1a). This B1-21 clone harbors a deletion within the coding region corresponding to the evolutionarily conserved RING domain of the human *BRCA1* gene (NM_007294.4) [28–30]. The N-terminal RING domain, disrupting E3 ubiquitin ligase activity and BRCA1–BARD1 complex formation, is essential for *BRCA1* function and is highly conserved among mammals [31]. Of the known pathogenic *BRCA1* mutations, ~10% are missense substitutions or in-frame insertion or deletion mutations located in the RING domain or C-terminal BRCT domain, although the vast majority are protein-truncating mutations resulting from premature stop codons [32, 33]. The missense variant c.181T>G (p.Cys61Gly) in the RING domain is also frequently detected in breast and ovarian cancers [34, 35].

Chromosome analysis revealed that the B1-21 carries 19 chromosomes, whereas the parental V79 and *BRCA2*-deficient V-C8 lines each carry 21 chromosomes (Fig. 1e). This chromosomal loss may have resulted from genomic instability arising during CRISPR/Cas9 editing or from compromised *BRCA1* function itself. We noticed that subpopulation of B1-21 cells contains mainly 19 chromosomes, reinforcing the notion that *BRCA1* deficiency induces genomic instability. B1-21 cells were re-cloned and confirmed strong chromosome number instability (Fig. 1f). Comparable chromosomal abnormalities have been observed in other *BRCA1*-deficient models [36, 37] and have also been reported in *BRCA2*-deficient cells [38] due to gross chromosomal rearrangement and centrosome amplification. Observing chromosomal instability in B1-21 cells may help elucidate how *BRCA1* deficiency leads to cancer development.

Comparative drug sensitivity profiling revealed distinct differences between the B1-21 and V-C8 mutants (Fig. 2). Although loss of either *BRCA1* or *BRCA2* conferred hypersensitivity to various DNA-damaging agents, the pattern of sensitivity differed between the two. Previous studies have shown that V-C8 cells are highly sensitive to camptothecin, with a *D*_10_ value ~10-fold lower than that of wild-type V79 cells [39]. Remarkably, the B1-21 (*BRCA1*-mutant) cells exhibited even greater sensitivity, with an IC_50_ value ~54-fold lower than wild-type cells and 10-fold lower than V-C8 cells (Table 1). This observation supports the concept that Topoisomerase I-induced DNA lesions are primarily repaired through HR, in which *BRCA1* plays a critical role in replication-fork stabilization and HR initiation [40]. Conversely, V-C8 cells exhibited higher sensitivity than B1-21 to interstrand cross-linking (ICL) agents such as cisplatin and mitomycin C, as well as to complex DSB inducers like etoposide and bleomycin (Table 1). This hypersensitivity likely reflects *BRCA2*’s essential role in HR, particularly its function in loading and stabilizing RAD51 filaments on single-stranded DNA, a process lost in V-C8 due to truncation of the RAD51-binding BRC repeat region [41].

PARP inhibitors are well established as targeted therapeutics for tumors deficient in *BRCA1* or *BRCA2*. In the present study, no significant differences were observed between *BRCA1*- and *BRCA2*-mutant cells in response to Olaparib, consistent with findings in a DT40-based isogenic model [42]. However, the B1-21 clone exhibited markedly stronger cytotoxicity to NU1025, one of the earliest PARP-1 inhibitors. Previous work has shown that different pharmacological PARP inhibitors display variable potency and sensitizing effects in *BRCA1*-positive breast cancer cells [43]. Moreover, studies using non-isogenic cell line collections have indicated that *BRCA1* or *BRCA2* mutations alone do not always predict increased sensitivity to PARP inhibitors [44]. In contrast, an isogenic mouse embryonic stem-cell model demonstrated that ABT-888 (veriparib), a potent PARP inhibitor, was more effective against *BRCA1* deficient cells [45]. Together, these findings highlight that various cellular factors beyond *BRCA* status contribute to PARP inhibitor responsiveness and underscore the importance of using an isogenic model system for mechanistic evaluation.

The known defects in HR repair and radiation-indued G2/M cell cycle checkpoint in *BRCA1* deficiency were confirmed in B1-21 cells (Figs 4 and 5) [46]. These defects were partial compared to V-C8 cells. Loss of *BRCA2* prevents proper RAD51 loading onto resected DNA ends, thereby abolishing HR activity [10]. Accordingly, these cells displayed elevated FANCD2 foci, reflecting hyperactivation of the Fanconi anemia (FA) pathway as a compensatory response to persistent DNA damage [47]. In contrast, as previously studies have shown that *BRCA1* mutants exhibit a decrease in RAD51 focus formation [48], and B1-21 cells also showed diminished RAD51 foci formation. Cell cycle analysis revealed a partial defect in the G2/M arrest in B1-21 cells, while V-C8 exhibited a more severe defect in the radiation-induced G2/M arrest (Fig. 5). Radiation-induced SCE relies on HR. In our study, there was no significant radiation-induced SCE formation in either B1-21 cells or V-C8 cells (Fig. 4c), consistent with prior observations that V-C8 cells are defective in mitomycin C-induced SCE formation [10].

Importantly, B1-21 cells lacked several of the phenotypes observed in V-C8. B1-21 presented elevated spontaneous chromatid breaks, but radiation did not induce chromatid break formation. V-C8 showed a significant increase in chromatid type aberrations following G1 irradiation (Fig. 4a). Failure to restart stalled replication forks likely results in fork collapse and chromatid breaks [49]. The marked increase in chromatid breaks after G2 irradiation (Fig. 4b), together with the persistent damage observed in the G2-PCC assay (Fig. 4d), indicates a substantial delay in chromatid break repair during the G2 phase in V-C8. The *BRCA2* mutation in V-C8 (biallelic truncating mutation in exon 15 and exon 16) eliminates the RAD51-binding domain, preventing filament assembly [11]. Although the hypomorphic *BRCA1* mutation in B1-21 may not fully impair its function, its heightened sensitivity to DNA-damaging agents demonstrates that BRCA1 is critically required for restarting stalled replication forks and repairing chromatid breaks during the G2 phase. While B1-21 exhibits a less severe repair defect than the BRCA2-null V-C8 cells, its genomic instability underscores the essential role of the BRCA1 RING domain in these pathways.

In conclusion, we successfully generated and characterized a *BRCA1* hypomorphic mutant that models clinically relevant loss-of-function mutations. The phenotypes of B1-21 align closely with previously reported *BRCA1*-deficient systems. This V79-based isogenic model of *BRCA1* and previously isolated *BRCA2* enables direct comparison of gene-specific vulnerabilities within an identical genetic background. This system provides a robust platform for dissecting gene-specific DNA repair mechanisms and identifying vulnerabilities relevant to targeted cancer therapy.

## Data Availability

All data generated or analyzed during this study are included in this published article.
